# Extrapulmonary *Pneumocystis jirovecii* infection in an advanced HIV-infected patient: A case report and literature review

**DOI:** 10.1186/s12879-023-08143-w

**Published:** 2023-03-29

**Authors:** Lalita Tancharoen, Soranart Muangsomboon, Patsharaporn T. Sarasombath, Nasikarn Angkasekwinai

**Affiliations:** 1 Division of Infectious Diseases and Tropical Medicine, Department of Medicine, Faculty of Medicine Siriraj Hospital, Mahidol University, 2 Wanglang Road, Bangkoknoi, Bangkok, 10700 Thailand; 2 Department of Pathology, Faculty of Medicine Siriraj Hospital, Mahidol University, Bangkok, Thailand; 3 Department of Parasitology, Faculty of Medicine Siriraj Hospital, Mahidol University, Bangkok, Thailand

**Keywords:** Extrapulmonary pneumocystosis, *Pneumocystis jirovecii*, HIV, Pneumocystis pneumonia, Paravertebral mass

## Abstract

**Background:**

*Pneumocystis jirovecii* infection is the most common opportunistic infection that causes pneumonia in human immunodeficiency virus (HIV)-infected patients; however, extrapulmonary *P. jirovecii* infection is extremely rare after the use of antiretroviral therapy. Here, we present the second reported case of paraspinal mass caused by *P. jirovecii* infection in an advanced HIV-infected patient.

**Case presentation:**

A 45-year-old woman presented with dyspnea on exertion, and significant weight loss within the preceding 4 months. Initial complete blood count (CBC) findings revealed pancytopenia with a hemoglobin (Hb) level of 8.9 g/dL, a white blood cell (WBC) count of 2180 cells/mm^3^ with 68% neutrophils, and a platelet count of 106,000 cells/mm^3^. Anti-HIV was positive with an absolute cluster of differentiation 4 (CD4) count of 16 cells/ mm^3^. A computed tomography scan of the chest revealed an enhancing soft tissue mass-like lesion at the right paravertebral region (T5-T10 level) and a thick-walled cavity lesion at the left lower lung. A CT-guided biopsy of the paravertebral mass was performed and histopathology revealed granulomatous inflammation consisting of dense aggregates of epithelioid cells and macrophages, and scattered foci of pink foamy to granular materials amidst the granulomatous inflammation. Gomori methenamine silver (GMS) staining revealed thin cystic-like structures (ascus) that were observed to be morphologically consistent with *P. jirovecii.* Molecular identification and DNA sequencing from the paraspinal mass was 100% identical to *P. Jirovecii*. The patient was successfully treated with oral trimethoprim-sulfamethoxazole for 3 weeks and antiretroviral therapy (ART) with tenofovir (TDF), lamivudine (3TC), and dolutegravir (DTG). A follow-up CT scan of the chest at 2 months after treatment showed a decrease in sizes of both the paravertebral mass and the cavitary lung lesion.

**Conclusions:**

Extrapulmonary pneumocystosis (EPCP) has become an extremely rare condition in HIV-infected patients after the widespread use of ART. EPCP should be considered in ART-naive HIV-infected patients suspected of having or diagnosed with *Pneumocystis jirovecii* pneumonia who present with atypical symptoms and/or signs. Histopathologic examination with GMS staining of affected tissue is necessary for the diagnosis of EPCP.

## Background

*Pneumocystis jirovecii* (*P. jirovecii*) infection remains one of the most prevalent opportunistic infections in patients with advanced human immunodeficiency virus (HIV) infection [1], and the prevalence was recently increasing among non-HIV-infected immunocompromised individuals, such as those with hematologic malignancy, transplantation recipients, and those receiving immunosuppressive agents [2]. The most common presenting symptom is subacute pneumonia. Extrapulmonary pneumocystosis (EPCP) is an extremely rare condition after the use of antiretroviral therapy. Here, we present the second reported case of paraspinal mass due to *P. jirovecii* in a patient with advanced HIV.

## Case presentation

**Fig. 1:**
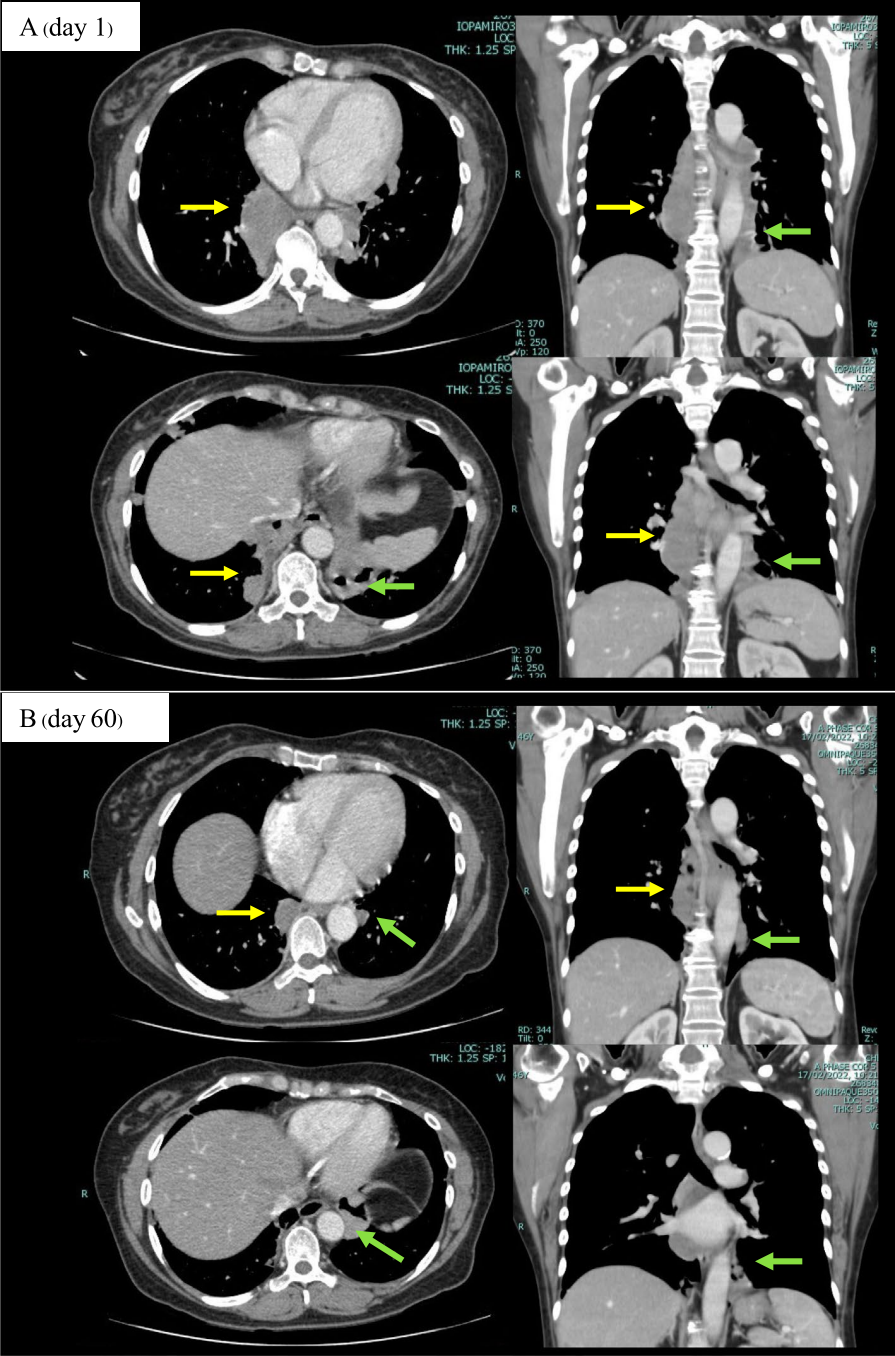
Computed tomography (CT) scan of the chest in transverse section on the left and coronal section on the right showed an enhancing soft tissue mass-like lesion (3.8 × 6.6 × 9.3 cm) at the right paravertebral region T5-T10 level (yellow arrows) and 3.1 × 4.6 cm thick-walled cavity lesion at the left lower lung (green arrows) **(A-day 1)** that decreased in size to 3.2 × 2.4 × 7.3 cm of right paravertebral soft tissue mass (yellow arrows) and 2.3 × 2.9 cm of left lower lung lesion (green arrows) after 2 months of initiating treatment **(B-day 60)**

**Fig. 2:**
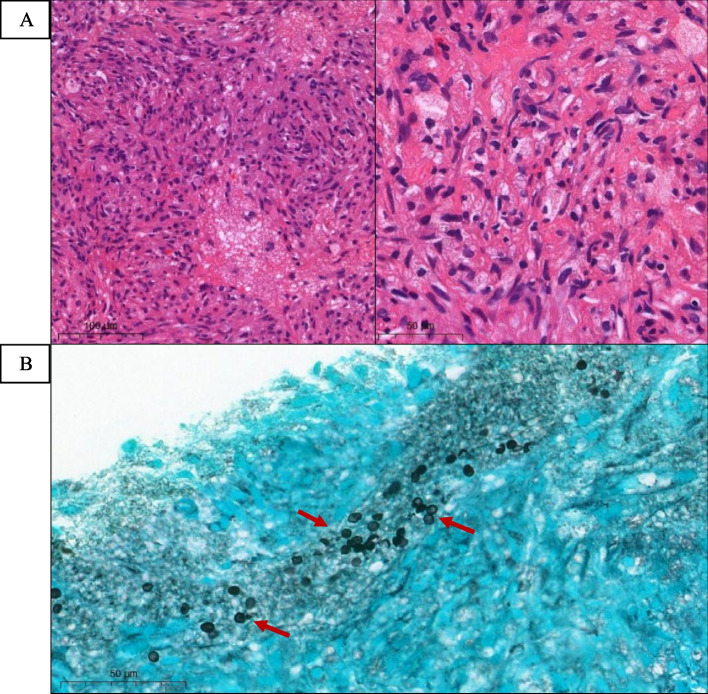
Histopathologic examination with hematoxylin-eosin (H&E) staining of right paravertebral mass revealed granulomatous inflammation consisting of dense aggregates of epithelioid cells and foamy macrophages mixed with some lymphocytes, plasma cells, few eosinophils, and fibroblastic proliferation. Scattered foci of pink foamy to granular materials can be observed amidst the granulomatous inflammation at magnification of 400x on the left and of 1000 x on the right (**A**). Gomori methenamine silver (GMS) staining revealed some thin cystic-like structures (red arrows) that were observed to be morphologically consistent with asci of *Pneumocystis jirovecii* at 1000 x magnification (**B**)

A previously healthy 45-year-old woman presented with dyspnea on exertion, anorexia, and marked weight loss (from 68 to 51 kg) within the preceding 4 months. She visited her primary care hospital several times and her initial complete blood count (CBC) revealed pancytopenia with a hemoglobin (Hb) level of 10.4 g/dL, a white blood cell (WBC) count of 2370 cells/mm^3^ (53% neutrophils, 21% lymphocytes, and 14% eosinophils), and a platelet count of 96,000 cells/mm^3^. Direct Coombs’ test (direct antiglobulin test) was positive (2+). Serum protein electrophoresis, immunofixation, and serum free light chain were all normal. Bone marrow examination revealed normocellular trilineage marrow with normal maturation and a slight to moderate increase in interstitial polytypic plasma cells suggestive of reactive plasmacytosis. She was diagnosed with autoimmune hemolytic anemia (AIHA) and was treated with prednisolone 30 mg/day for 2 weeks. The dose of prednisolone was then increased to 60 mg/day due to the progression of anemia. Trimethoprim-sulfamethoxazole (TMP/SMX) (80/400) at a dosage of 2 tablets 3 times/week was given for primary prophylaxis against *Pneumocystis jirovecii* pneumonia (PCP) since starting treatment with prednisolone. Further investigations were performed to investigate the secondary cause of AIHA such as hematologic malignancies particularly lymphoma. Therefore, a computed tomography (CT) scan of the whole abdomen was performed which revealed a matted lobulated soft tissue mass-like lesion (4.0 × 2.7 × 8.8 cm) at the paravertebral region (T6-T9), and no significant intra-abdominal lymphadenopathy or hepatosplenomegaly. She was then referred to our national tertiary referral center so that the identified lesion could be further investigated. Other reported complaints included a non-productive cough and fatigue for 2 weeks prior to referral. Her initial physical examination at our center revealed no fever, no dyspnea or tachypnea, and an oxygen saturation rate of 98% at room air. She was moderately pale, and had oral thrush at the buccal mucosa and pruritic papular eruption on both legs. Other examinations were unremarkable. Oral candidiasis was recognized. Initial CBC revealed pancytopenia with an Hb level of 8.9 g/dL (normal range, 12.0–14.9 g/dL), a WBC count of 2180 cells/mm^3^ (normal range, 4400–10,300 cells/mm^3^), with 68% neutrophils, 12% lymphocytes, and 5% eosinophils, and a platelet count of 106,000 cells/mm^3^ (normal range, 179,000–435,000 cells/mm^3^). Blood test for anti-HIV was positive with an initial cluster of differentiation 4 (CD4) count of 16 cells/L (3.49%) and HIV viral load of 304,000 copies/mL. Serum cryptococcal antigen test was negative. CT scan of the chest showed an enhancing soft tissue mass-like lesion (3.8 × 6.6 × 9.3 cm) at the right paravertebral region (T5-T10 level), and a 3.1 × 4.6 cm thick-walled cavity lesion at the left lower lung (Fig. 1A). Fiberoptic bronchoscopy with bronchoalveolar lavage (BAL) was performed after receiving TMP/SMX prophylaxis for 3 months. The results of BAL fluid analysis were, as follows: WBC count of 126 cells/mm^3^ (75% lymphocytes, 21% macrophage, and 3% neutrophils). BAL fluid cultures for bacteria, mycobacteria, and fungus were all negative. BAL immunofluorescence assay (IFA) for *P. jirovecii* was negative, and BAL cytology was negative for malignancy. A CT-guided biopsy of the paravertebral mass was performed and histopathology revealed granulomatous inflammation consisting of dense aggregates of epithelioid cells and macrophages, and scattered foci of pink foamy to granular materials amidst the granulomatous inflammation (Fig. 2A). Gomori methenamine silver (GMS) staining revealed some thin cystic-like structures that were observed to be morphologically consistent with *P. jirovecii* ascus-like cystic form (Fig. 2B). Cultures of paraspinal tissue for bacteria and mycobacteria were all negative. The serum BDG level was not tested as it is not available in our hospital and fungal culture for *P. jirovecii* was not performed due to the lack of a stable ex vivo culture system. Quantitative PCR targeted mitochondrial large subunit ribosomal RNA (mtLSU rRNA) was used for the detection of *P. jirovecii* in both BAL fluid and paraffin-embedded tissue of the paraspinal mass using the protocol as previously described [3, 4]. *P. jirovecii* was detected only in the paraspinal specimen. Conventional PCR targeted a partial mtLSU rRNA from the paraspinal mass was further performed as previously described [3] and subjected to DNA sequencing to confirm the causative species. The DNA sequence from this patient was 100% identical to *P. jirovecii* (GenBank accession No. MW530527.1) and was submitted to GenBank under accession. A confirmed diagnosis of extrapulmonary *P. jirovecii* presenting as a paraspinal mass and *P. jirovecii* pneumonia (PCP) was established. The patient was treated with trimethoprim-sulfamethoxazole (TMP/SMX) at dose of 15 mg/kg/day of TMP and 75 mg/kg/day of SMX for a total of 3 weeks, which was followed by continued secondary prophylaxis using the same medication. Antiretroviral therapy (ART) with dolutegravir (DTG)/tenofovir (TDF)/lamivudine (3TC) (50/300/300) was started and the prednisolone from the primary hospital was discontinued. The patient’s symptoms including non-productive cough, dyspnea on exertion, loss of appetite, and weight loss were dramatically improved after treatment, she had no cough, no anorexia or fatigue, and she gained 4 kg in body weight in one month. A CT scan of the chest performed at 2 months after starting treatment showed a decrease in the size of the paravertebral soft tissue mass and the left lower lung cavitary lesion (Fig. 1B). Her absolute CD4 count increased to 202 cells/mm^3^ (10.63%), and her HIV viral load became undetectable. She had no recurrent symptoms after a year of follow-up.

## Discussion

*Pneumocystis jirovecii* pneumonia (PCP) develops mainly in patients with advanced HIV infection, newly diagnosed patients, and those not using antiretroviral therapy (ART). Dry cough, subacute fever, dyspnea, desaturation, and interstitial infiltration with ground glass opacity from chest imaging are presenting signs and symptoms that increase the suspicion of a diagnosis of PCP. Extrapulmonary pneumocystosis (EPCP) is an extremely rare condition [5] after the wide availability use of ART. Approximately 50–80% of reported EPCP occurred in advanced HIV-infected patients who did not receive TMP/SMX prophylaxis or received aerosolized pentamidine as prophylaxis [6]. EPCP was also reported in other types of immunocompromised patients such as patient with connective tissue disease, solid organ transplant or hematopoietic stem cell transplant recipients and those with hematologic malignancy who receive immunosuppressive drugs [6, 7]. In this article, we describe the second reported case of an advanced HIV patient with EPCP presenting as a paravertebral mass [8]. Our patient was diagnosed EPCP while taking a high dose of prednisolone and a double strength of TMP/SMX three times per week for PCP prophylaxis. However, this does not imply that TMP/SMX prophylaxis is ineffective because paravertebral soft tissue mass was already found on CT scan of the whole abdomen while starting TMP/SMX prophylaxis.

The incidence of EPCP among HIV-infected patients was estimated to be 0.06–2.5% [7]. The clinical presentation of EPCP depends on the organ(s) involved, and can present with or without coexisting pulmonary infection [6, 7]. Approximately 60–64% of patients with EPCP had multiple organ involvement at either contiguous or non-contiguous sites. Postmortem evidence revealed the routes of dissemination to be hematogenous, lymphatic, or direct spreading from an adjacent organ [6, 9]. From 1954 to 1997, there were 90 and 16 cases of EPCP reported in HIV-infected and non-HIV-infected patients, respectively. Among HIV-infected patients, EPCP was the most prevalent in advanced disease and more frequently presented with multiple organ involvement. Ocular or ear was the most common organ involved in those with single organ involvement and was associated with a better prognosis [6]. In contrast, non-HIV-infected patients with EPCP had non-specific symptom(s), more dissemination, and had worse outcome with > 50% diagnosed postmortem. Other common organs involved include the lymph nodes, spleen, liver, and bone marrow. Less commonly involved organs include the pleura, trachea, diaphragm, meninges, cerebral cortex, gastrointestinal tract, pancreas, kidney, heart, adrenal gland, pituitary gland, parathyroid gland, and spine [6]. Although more than 100 cases of EPCP were reported before 1997, only 5 cases of adult EPCP were reported after that time point and all 5 of those patients were HIV positive [8, 10–13]. Of those 5 patients, 3 were treatment-naïve with a CD4 count of less than 50 cells/mm^3^ [8, 10, 12], and 4 out of 5 had multiple organ involvement. One of those 3 treatment-naïve cases had paravertebral soft tissue involvement like the patient described in the present report [8]. Only one patient who was on ART, with a CD4 count of 180 cells/mm^3^ had EPCP restricted to only the ear [11] (Table 1).

**Table 1:** The demographic and clinical characteristics of 5 HIV-infected patients who developed extrapulmonary *Pneumocystis jirovecii* infection and that had their cases reported after 1997

	Case 1 [11]	Case 2 [8]	Case 3 [12]	Case 4 [13]	Case 5 [10]
Age / gender	38 years / male	46 years / male	39 years / male	31 years / male	39 years / male
Treatment status	ART	Treatment naive	Treatment naive	Poor adherence	Treatment naïve
CD4 count (cells/mm^3^)	180	1.8	20	< 200	36
Duration of symptom onset	1 month	6 months	2 weeks	3 weeks	1 year
Clinical presentation	Left ear otorrhea, progressive deafness	Prolonged fever, dyspnea, abdominal pain	Productive cough with dyspnea	Diarrhea, abdominal pain and fever	Decreased appetite, abdominal fullness, weight loss, anemia
Organ involvement	Left external-middle ear to middle cranial fossa	Liver, pancreas, spleen, vertebrae, paravertebral soft tissue, pleura, jejunum	Lung, liver, intra-abdominal lymph nodes, jejunum	Lung, liver, spleen	Spleen, liver, bilateral adrenal gland
Investigation	Mass biopsy for histologic diagnosis	Vertebral lesion biopsy for histologic diagnosis	Sputum PCR – positive, liver biopsy: organism not found	Spleen and lung biopsy for histologic diagnosis	Spleen biopsy for histologic diagnosis – PCR positive
Treatment	IV TMP and oral dapsone for 3 weeks	TMP/SMX for 3 weeks	TMP/SMX for 3 weeks	TMP/SMX	IV TMP/SMX for 3 weeks then IV pentamidine for 3 weeks, surgicaldrainage
Outcome	Full recovery	Improved	Improved	Improved	Full recovery

*Abbreviations*: *HIV* human immunodeficiency virus, *ART* antiretroviral therapy, *CD4* cluster of differentiation 4, *mm*^*3*^ millimeters cubed, *PCR* polymerase chain reaction, *PCP Pneumocystis jirovecii* pneumonia, *IV* intravenous, *TMP/SMX* trimethoprim-sulfamethoxazole

A diagnosis of EPCP can be established via demonstration of the eosinophilic foamy exudates in which *P. jirovecii* cystic form (ascus) or trophic form are embedded in affected tissue by Gomori methenamine silver (GMS) staining or periodic acid-Schiff (PAS) staining. The finding of GMS staining with spherical, helmet, or crescent non-budding ascus cyst-like structure, approximately 4–8 μm in size differs from *Histoplasma* spp. which described as oval budding yeast with smaller in sizes of 1–4 μm [14]. Notably, a diagnosis of EPCP can be delayed due to uncommon presentation of EPCP and delayed HIV diagnosis, such as in our case, which was referred for investigation of a paraspinal mass before our patient’s HIV status was investigated. Detection of *P. jirovecii* in a pulmonary specimen, such as BAL fluid or lung biopsy, with Giemsa or immunofluorescent stains is also useful when considering EPCP in the differential diagnosis. Histopathologic examination with GMS staining is generally sufficient for detection of *P. jirovecii*; however, detection of *P. jirovecii* by PCR, which is more sensitive than GMS staining, may be useful to confirm the diagnosis, especially in EPCP patients who present with uncommon manifestation. However, BAL fluid PCR was negative for *P. jirovecii* in our patient. It could probably be explained by several reasons. First, our patient received the prophylaxis dose of TMP/SMX for 3 months before performing bronchoscopy, which might have cleared *P. jirovecii* DNA, resulting in false negative BAL fluid PCR [15, 16]. In addition, the BAL specimen was sent for PCR testing after the IFA for *P. jirovecii* yielded a negative result, which means that the specimen might not properly preserved for DNA testing.

There is currently no specific recommendation for the treatment of EPCP. Previous EPCP cases among HIV-infected patients were treated with TMP/SMX as the first-line regimen for PCP [10]. Approximately 80% of EPCP cases series were treated with TMP/SMX. Other drugs, such as dapsone plus trimethoprim, primaquine plus clindamycin, atovaquone, or intravenous pentamidine have been used when patients could not tolerate or did not improve after treatment with TMP/SMX [6]. The duration of treatment varied from 3 to 4 weeks [8, 11, 12]; however, prolonged duration of antibiotics with surgical drainage would be needed in cases of large and multiple intra-abdominal organ abscesses [10]. The prognosis is poor with > 50% mortality among those with multiple organ involvement [6]. Early initiation of effective ART is important for restoring immune status in HIV-infected patients. Our patient had clinical improvement after treatment with 3 weeks of TMP/SMX and early ART initiation.

In conclusion, EPCP has become an extremely rare disease in HIV-infected patients after the widespread use of ART. EPCP should be considered in ART-naive HIV-infected patients who are suspected of having or diagnosed with PCP and in patients with atypical symptoms and/or signs. Histopathologic examination with GMS staining of affected tissue is necessary for the diagnosis of EPCP.

## Data Availability

All data during this study are included in this article.

## Abbreviations

3TC: Lamivudine
AIDS: Acquired immunodeficiency syndrome
AIHA: Autoimmune hemolytic anemia
ART: Antiretroviral therapy
BAL: Bronchoalveolar lavage
CBC: Complete blood count
CD4: Cluster of differentiation 4
CT: Computed tomography
DTG: Dolutegravir
EPCP: Extrapulmonary pneumocystosis
GMS: Gomori methenamine silver
Hb: Hemoglobin
IFA: Immunofluorescence assay
PAS: Periodic acid-Schiff
PCR: Polymerase chain reaction
PCP: *Pneumocystis jirovecii* pneumonia
TDF: Tenofovir
TMP/SMX: Trimethoprim-sulfamethoxazole
WBC: White blood cell
